# NF-κB inhibitor with Temozolomide results in significant apoptosis in glioblastoma via the NF-κB(p65) and actin cytoskeleton regulatory pathways

**DOI:** 10.1038/s41598-020-70392-5

**Published:** 2020-08-07

**Authors:** Naze G. Avci, Sadaf Ebrahimzadeh-Pustchi, Yasemin M. Akay, Yoshua Esquenazi, Nitin Tandon, Jay-Jiguang Zhu, Metin Akay

**Affiliations:** 1 Department of Biomedical Engineering, University of Houston, 3517 Cullen Blvd, Houston, TX 77204-5060 USA; 2 UTHealth Neurosurgery, McGovern Medical School, Memorial Hermann at Texas Medical Center, The University of Texas Health Science Center at Houston, Houston, TX 77030 USA

**Keywords:** Cancer microenvironment, Cancer therapy

## Abstract

Glioblastoma (GBM) is the most malignant brain tumor characterized by intrinsic or acquired resistance to chemotherapy. GBM tumors show nuclear factor-κB (NF-κB) activity that has been associated with tumor formation, growth, and increased resistance to therapy. We investigated the effect of NF-κB inhibitor BAY 11-7082 with Temozolomide (TMZ) on the signaling pathways in GBM pathogenesis. GBM cells and patient-derived GBM cells cultured in 3D microwells were co-treated with BAY 11-7082 and TMZ or BAY 11-7082 and TMZ alone, and combined experiments of cell proliferation, apoptosis, wound healing assay, as well as reverse-phase protein arrays, western blot and immunofluorescence staining were used to evaluate the effects of drugs on GBM cells. The results revealed that the co-treatment significantly altered cell proliferation by decreasing GBM viability, suppressed NF-κB pathway and enhanced apoptosis. Moreover, it was found that the co-treatment of BAY 11-7082 and TMZ significantly contributed to a decrease in the migration pattern of patient-derived GBM cells by modulating actin cytoskeleton pathway. These findings suggest that in addition to TMZ treatment, NF-κB can be used as a potential target to increase the treatment’s outcomes. The drug combination strategy, which is significantly improved by NF-κB inhibitor could be used to better understand the underlying mechanism of GBM pathways in vivo and as a potential therapeutic tool for GBM treatment.

## Introduction

Glioblastoma multiforme (GBM) is the most malignant primary brain tumor in the central nervous system. Current standard of care therapy includes surgery followed by radiotherapy and concomitant and adjuvant chemotherapy with the alkylating agent Temozolomide (TMZ), which provides survival benefits for patients with GBM^1^. However, even with the advances in surgical resection combined with TMZ therapy and irradiation, the prognosis for newly diagnosed GBM patients remains poor. In fact, due to its rapid proliferation, increased invasion and migration capacity and chemoresistance to the alkylating agents the median survival is only 14.6 months with the ‘Stupp’ regimen (radiation with daily TMZ × 4–6 weeks followed by cyclic TMZ)^2^ and 5-year survival rate is less than 6%, which is the lowest long-term survival rate of malignant brain tumors^3–5^. TMZ methylates DNA at the O^6^ positions of guanine and DNA repair enzyme O^6^-methylguanine methyltransferase (MGMT) removes alkyl groups from O^6^ position of guanine in DNA making cells resistant to TMZ^6^. Therefore, new therapies are necessary to prevent cell proliferation and induce apoptosis for GBM patients.

Nuclear factor-kappa B (NF-κB) is a regulatory transcription factor of the Rel gene family including p50, c-Rel, RelB, or p65 subunits. It is involved in the control of tumor cell proliferation, migration, immune response and apoptosis^7–10^. Studies have shown that NF-κB gene was involved in the regulation pathways of different cancer types such as thyroid cancer, head and neck squamous cell carcinoma and colorectal cancer^7,11–13^. Increased activation of NF-κB has also been identified in GBM tumors, where the expression of NF-κB was much higher in GBM tissue compared with non-GBM tissue^14,15^. NF-κB also promotes chemoresistance to TMZ and regulates MGMT activity in GBM by promoting MGMT gene expression through NF-κB binding sites within the MGMT promoter^16^. NF-κB inhibitors such as parthenolide do not completely eradicate tumors, therefore, they are mostly used in combination with other drugs^17^. When used in combination with TMZ, NF-κB inhibitor parthenolide has been shown to activate mitochondrial apoptosis signaling in U87MG and U373 GBM cells, which lead to cell death^18^ and had a combined effect on cell cytotoxicity in LN18 and T98G glioma cells^19^. NF-κB inhibitor CBL0137 has been shown to bind DNA leading the functional inactivation of the Facilitates Chromatin Transcription (FACT) complex, a chromatin remodeling complex regulating transcription, replication, and DNA repair^20,21^. In vitro evaluation of the CBL0137 on FACT, p53 and NF-κB has been done using U87MG and A1207 GBM cells. It was shown that CBL0137 induced loss of chromatin-unbound FACT, activated p53 and inhibited NF-κB dependent transcription^21^. In vivo studies showed that CBL0137 was effective in increasing survival rates in TMZ-resistant orthotopic mouse models^21^. Moreover, Wang et al. indicated that NF-κB inhibitor BAY 11-7082 suppresses the expression of MGMT and enhances the TMZ-induced apoptosis in TMZ resistant U251 cells^22^. However, there is still a lack of characterization of the precise pattern of NF-κB activation in combination with TMZ in GBM cell populations that have been surgically resected from patients.

In vitro and in vivo identifications and validations of molecular targets of GBM are important as they can progress into clinical studies. Studies reported that combining multiple gene targets may prevent tumor growth and improve the treatment strategy for GBM^23–27^. Both Bay 11-7082 and TMZ exert anti-tumoral activities individually in different tumor types^28–30^. Therefore, in this study, we aimed to analyze functionally the combined effect of Bay 11-7082 and TMZ in different GBM cells. For this purpose, first we used our 3D PEGDA-based hydrogel microwell platform^31–34^ to provide reliable preclinical models that can recapitulate in vivo features of the GBM tumors. We cultured GBM cells (U87 and LN229) and patient-derived GBM cells in 3D microwells for a more precise and personalized treatment approach. We then treated GBM cells with Bay 11-7082 and TMZ in combination or alone. Our results indicated that the co-treatment of Bay 11-7082 and TMZ significantly reduced cell viability in all three cell lines in correlation with a significant decrease in the spheroid size. The levels of NF-κB protein and its subunits p65 and p50 were also significantly decreased compared with the control and single drug applications. Similar decreases in the cell viability and protein levels were observed in all three GBM cells. Tumor biopsy samples could give more realistic information about how tumors respond to drugs when they are used for in vitro or in vivo studies^35–37^. Therefore, we decided to continue our experiments with only using our patient-derived GBM cells. We treated patient-derived GBM cells with Bay 11-7082 and TMZ or alone and analyzed specific cellular proteins along with their post-translational modifications via reverse-phase protein arrays (RPPA) to elucidate the mechanism of action of the proteins^38,39^. We observed that several cell signaling pathways including cell metabolism, proliferation, apoptosis were significantly affected by the combination of the drugs, which were consistent with the literature^40,41^. Furthermore, our RPPA data revealed that there was a significant change in the modulation of actin cytoskeleton and following experiments including western blot analysis for the expression of FAK protein and wound healing assay for cell migration patterns confirmed the RPPA results. We observed a significant decrease in both actin fluorescence intensity and migration pattern in the co-treated patient-derived GBM cells. To the best of our knowledge, the effect of co-treatment of Bay 11-7082 and TMZ has never been studied previously on the actin modulation of patient-derived GBM cells. These results suggested that Bay 11-7082 and TMZ induced alteration in the actin filament organization by reducing the level of focal adhesion protein, which might implicate in cell apoptosis. The effect of Bay 11-7082 with TMZ necessitates further exploration to better understand its mechanism of action in GBM and potential therapeutic tools for GBM treatment.

## Results

### Co-treatment of Bay 11-7082 and TMZ reduced viability of GBM cells

**Figure 1:**
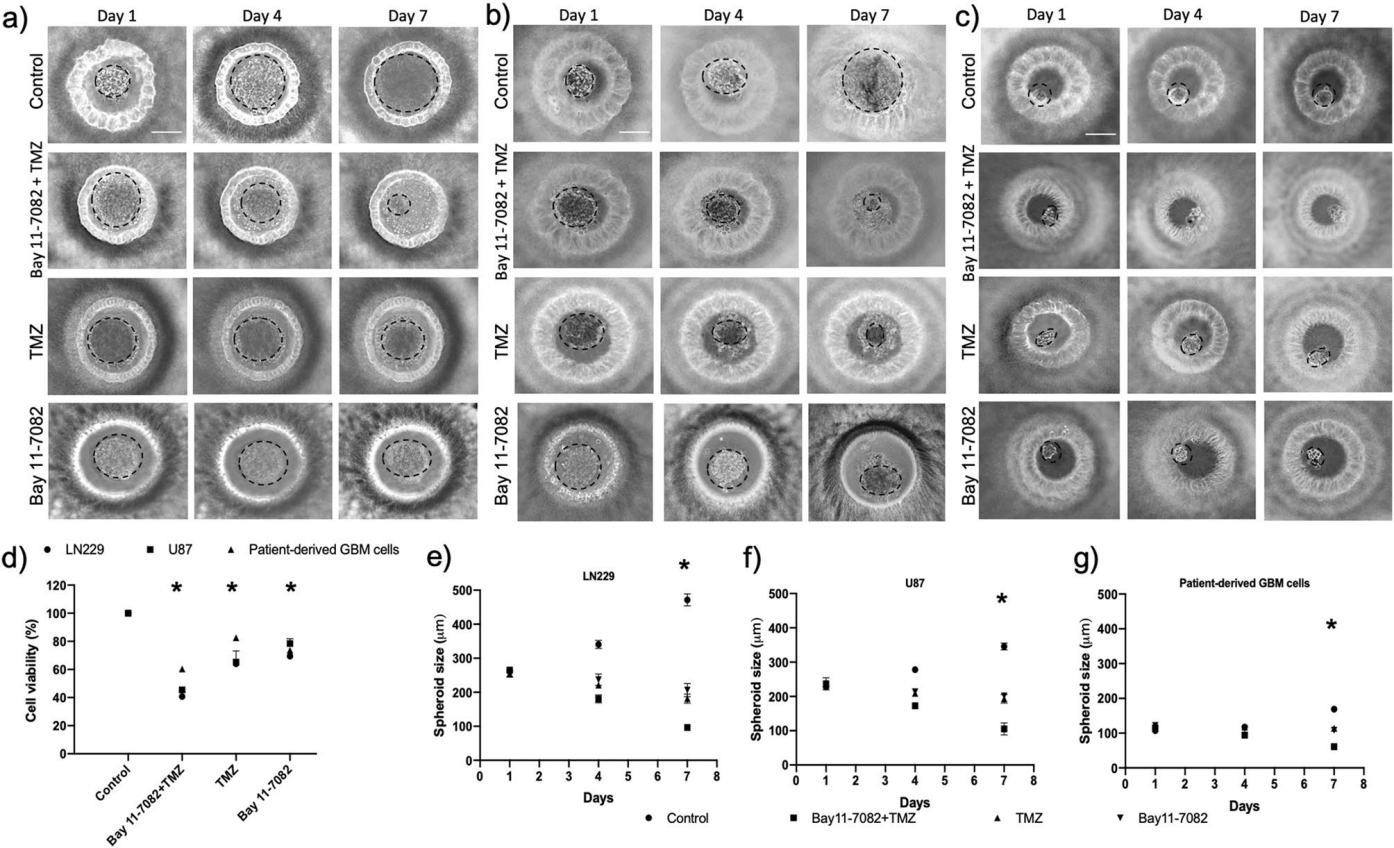
Representative images of the GBM tumor cells cultured in the PEGDA microwells. (**a**–**c**) LN229, U87 and patient-derived GBM cells were cultured in the microwells for 7 days, respectively. After day 7, Bay 11-7082 and TMZ were applied either alone or in combination onto the cell spheroids. Control group did not receive any treatment. The cells were cultured with or without drugs additional 7 more days. The images were taken on Day 1, Day 4 and Day 7 after the drug application to observe the disruption in the spheroids. Dotted black lines represent the edge of the tumor spheroid. Scale bars, 200 µm. (**d**) Bar graph showing trypan blue staining for cell viability of LN229, U87 and patient-derived GBM cells. (**e**–**g**) Spheroid size quantification was done using ImageJ for LN229, U87 and Patient-derived GBM cells, respectively. Two-tailed t-test followed by Wilcoxon test were done (GraphPad Prism v5). Data represent the mean ± SD of three biological replicates. * *p* < 0.05 and ** *p* < 0.01.

We used our previously published data to select the most effective drug concentrations for this study^42^. We cultured LN229, U87 and patient-derived cells in the microwells for 7 days where they formed 3D spheroids and we added 4.5 µM of Bay 11-7082 and 300 µM of TMZ in combination or alone. Then, we cultured the spheroids for 7 more days with or without drug. Control group did not receive any treatment. The cell viability assay was performed on day 7 after drug administration. The results showed that the co-treatment significantly reduced cell viability of GBM cells LN229 and U87 and patient-derived GBM cells cultured in 3D PEGDA microwells, respectively as shown in Fig. 1a-c. When they were used alone, TMZ reduced cell viability to 66%, 59% and 82% (*p* < 0.05) and Bay 11-7082 reduced cell viability to 69%, 78%, and 73% in LN229, U87 and patient-derived GBM cells, respectively compared to control groups (Fig. 1d). However, when they were used in combination, the viability of the cells significantly decreased to 42%, 45% and 60% in LN229, U87 and patient-derived GBM cells, respectively compared to control groups (*p* < 0.01) (Fig. 1d). Tumor cells are generally less sensitive to drug treatments in 3D cultures than in 2D cultures^43,44^. This could reflect reduced compound access or differences in the response to cell death. To confirm that co-treatment was more effective compared to single drug use, we quantified the size of the spheroids using ImageJ^45^. Our data showed that after 7 days of drug treatment, the spheroids’ sizes were significantly reduced in the co-treatment by 71%, 77% and 75% in LN229 (Fig. 1e), U87 (Fig. 1f) and patient-derived GBM cells (*p* < 0.01) (Fig. 1g), respectively compared to control group (*p* < 0.05). When we compared the spheroids’ sizes of the co-treatment with TMZ alone, there was a reduction of 57%, 51% and 40% in LN229, U87 and patient-derived GBM cells, respectively (*p* < 0.05). Finally, the spheroids’ sizes of the co-treatment compared with Bay 11-7082 alone showed a decrease of 37%, 49% and 40% in LN229, U87 and patient-derived GBM cells, respectively.

### Suppression of NF-κB activity in GBM cells by co-treatment of Bay 11-7082 and TMZ

**Figure 2:**
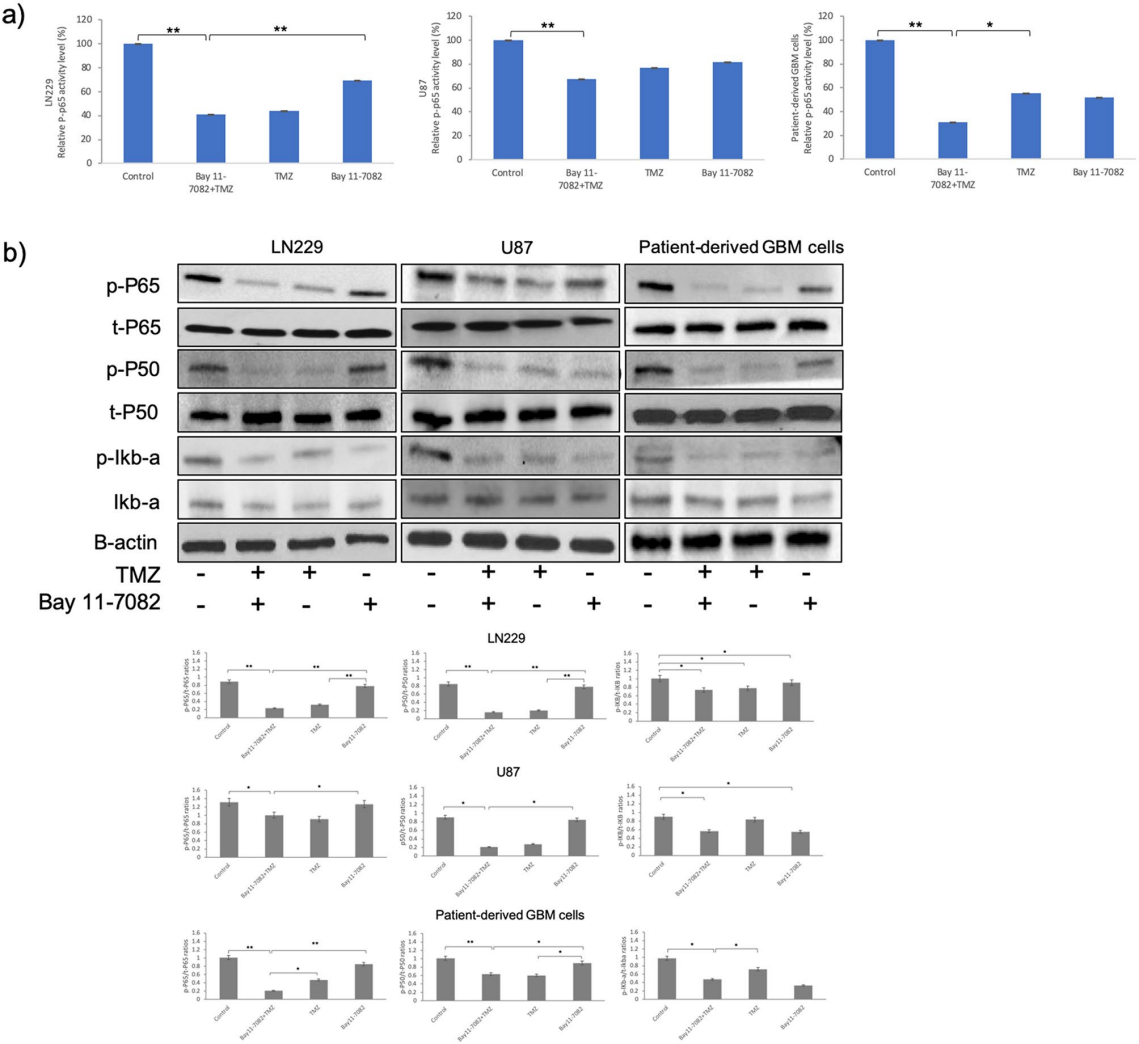
NF–kB activity in LN229, U87 and patient-derived GBM cell lines. (**a**) NF–kB p65 subunit activity in LN229, U87 and patient-derived GBM cell lines, respectively. The cells cultured with or without drugs for 7 days were collected from the microwells and subjected to ELISA. Data represent the mean ± SD of three biological replicates. * *p* < 0.05 and ** *p* < 0.01. (**b**) Representative immunoblots. LN229, U87 and patient-derived GBM cells were cultured with or without drugs for 7 days, lysed and immunoblotted with the indicated antibodies. Quantification of the fold-changes in protein levels (bottom panel). Data were normalized to B-actin. Data represent the mean ± SD of three biological replicates. * *p* < 0.05, ** *p* < 0.01.

As a readout of NF-kB activity after drug treatment, we first quantitatively assessed the cytoplasmic activation of phosphorylated NF-κB p65 subunit in both treated and untreated groups in all GBM cells. NF-κB p-p65 subunit activity was observed in the control groups of all three GBM cells (Fig. 2a). NF-κB p-p65 subunit activity decreased to 43%, 76% and 55% when TMZ applied alone and 69%, 81% and 52% when Bay 11-7082 was applied alone in LN229, U87 and patient-derived cells, respectively. However, the decrease in NF-κB p-p65 subunit was reduced to 40%, 67%, 31% when LN229, U87 and patient-derived cells, respectively were co-treated (*p* < 0.01) (Fig. 2a). Bay 11-7082 specifically inhibits NF-κB activation by blocking phosphorylation of IκB-α^46^. In independent experiments, we analyzed the abundance of phosphorylated NF-κB p65, NF-κB p50 and IκB-α in all three GBM cells. Qualitative and quantitative western blot analysis revealed that the exposure to Bay 11-7082 with TMZ significantly down-regulated the abundance of NF-κB p65, NF-κB p50 and IκB-α compared with control and Bay 11-7082 or TMZ alone (Fig. 2b). Please note that loading controls were used for each experiment, but only the representative loading control for *p* and t-P65 and *p* and t-P50 was presented (Fig. 2b). The cell viability assay, cells’ size and protein expressions in all three GBM cells revealed similar results without any dramatic change. Therefore, considering the importance of using patient-derived tumor cells to elucidate the mechanism of drugs and respective signaling pathways^35–37^, we further continued our experiments using patient-derived GBM cells.

### Apoptosis was promoted by co-treatment of Bay 11-7082 and TMZ

**Figure 3:**
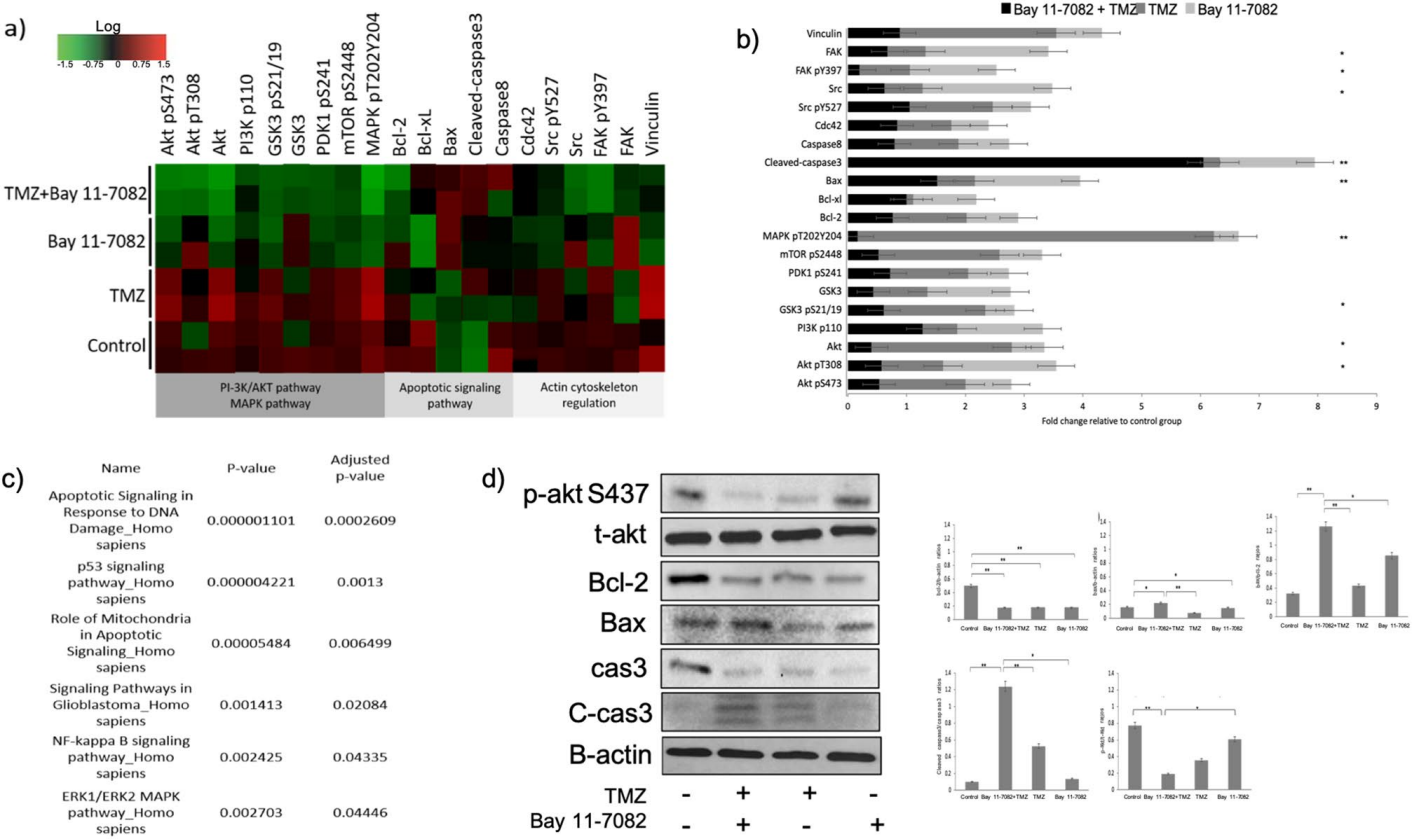
The effect of Bay 11-7082 and TMZ on signaling pathways in patient-derived GBM cells. (**a**) Heat map presentation of RPPA analysis showing the changes in the protein expression. RPPA was performed on lysates treated with Bay 11-7082 and TMZ alone or in combination. All relative protein level data points were normalized to the control group. Red and green indicate up and down regulations, respectively in the heat map. The samples were run in duplicate (n = 3). (**b**) Fold change of the selected proteins relative to the control group via RPPA. Data represent the mean ± SD of two biological replicates. (* *p* < 0.05, ** *p* < 0.01, Wilcoxon rank sum test). (**c**) Analysis of down-regulated RPPA proteins shows a significant activation in numerous Enrichr KEGG pathways. The pathways were sorted by *p* value ranking. (**d**) Representative immunoblot validation of significantly altered proteins involved in different KEGG pathways. Patient-derived GBM cells were cultured with or without drugs for 7 days, lysed and immunoblotted with the indicated antibodies. Quantification of the fold-changes in protein levels (right panel). Data were normalized to B-actin. Data represent the mean ± SD of three biological replicates. * *p* < 0.05, ** *p* < 0.01.

RPPA technology is designed for multiplexed, antibody-based relative quantification where each array is tested with a validated antibody specific to a particular protein along with their particular post-translational modifications^47^. In the attempt to elucidate the mechanism of action of Bay 11-7082 with TMZ by which NF-κB subunits were modulated and to identify downstream signaling molecules, we employed RPPA platform using our drug treated or untreated patient-derived GBM cells. RPPA results showed that many oncogenic pathways were altered by the drug treatments but more specifically by the co-treatment (Fig. 3a). Decreased expression of NF-κB was not only associated with changes in the NF-κB pathway but also with apoptosis, cell metabolism and proliferation, which were confirmed by the analysis of down-regulated RPPA proteins in Enrichr KEGG libraries^48,49^ (Fig. 3c) (*p* < 0.05).

One of the specific pathways given by RPPA was apoptosis. Apoptosis is one of the important mechanisms that regulates cell death and suppress tumorigenesis. Studies have demonstrated that Bcl-2 family proteins can positively and negatively regulate apoptosis by regulating anti-apoptotic protein Bcl-2 and pro-apoptotic protein Bax^40,50^. Our RPPA data using patient-derived GBM cells showed that the fold change of Bcl-2 relative to control was 0.71, 1.26, 0.88 times higher in co-treated group, TMZ alone, Bay 11-7082 alone, respectively (Fig. 3b). In order to further confirm whether the expression of these proteins were down-regulated by the co-treatment, we performed western blot analysis. Our results showed a similar decrease in Bcl-2 protein expression in the co-treatment compared with the control and single drug treatment (Fig. 3d). In contrast, Bax protein fold change relative to control was 1.61, 0.64, 1.41 times higher in co-treated group, TMZ alone, Bay 11-7082, respectively, where we observed a significant increase after the co-treatment of Bay 11-7082 with TMZ compared with the control (*p* < 0.01) (Fig. 3b). Bcl-2/Bax ratio is a key indicator in susceptibility of the cells to apoptosis. Western blot results confirmed the change in Bcl-2/Bax ratio in the co-treatment compared with the control group and single drug treatment (Fig. 3d). Our RPPA data also showed a significant increase in the cleaved-caspase protein fold change relative to control, 6.05 times higher in the co-treatment compared with 0.27 times higher in TMZ alone and 1.61 times higher in Bay 11-7082 alone (*p* < 0.01) (Fig. 3b). To confirm if co-treatment triggered apoptosis correlated with caspase activation, we performed western blot analysis with pro-caspase-3 (cas3) and cleaved-caspase-3 (C-cas3). We observed that Bay 11-7082 and TMZ induced apoptosis was associated with cas3 (Fig. 3d). Please note that loading controls were used for each experiment, but only the representative loading control for Bax, cas3 and C-cas3 was presented (Fig. 3d). Moreover, another important mechanism of NF-κB activation in GBM regulates through AKT phosphorylation of IκB. Our RPPA data showed relative fold changes of 0.57, 1.04, 1.92 in the co-treated group, TMZ alone and Bay 11-7082 alone, respectively (*p* < 0.05) (Fig. 3b). The western blot results also confirmed a significant decrease in the abundance of AKT pT308. (Fig. 3d).

**Figure 4:**
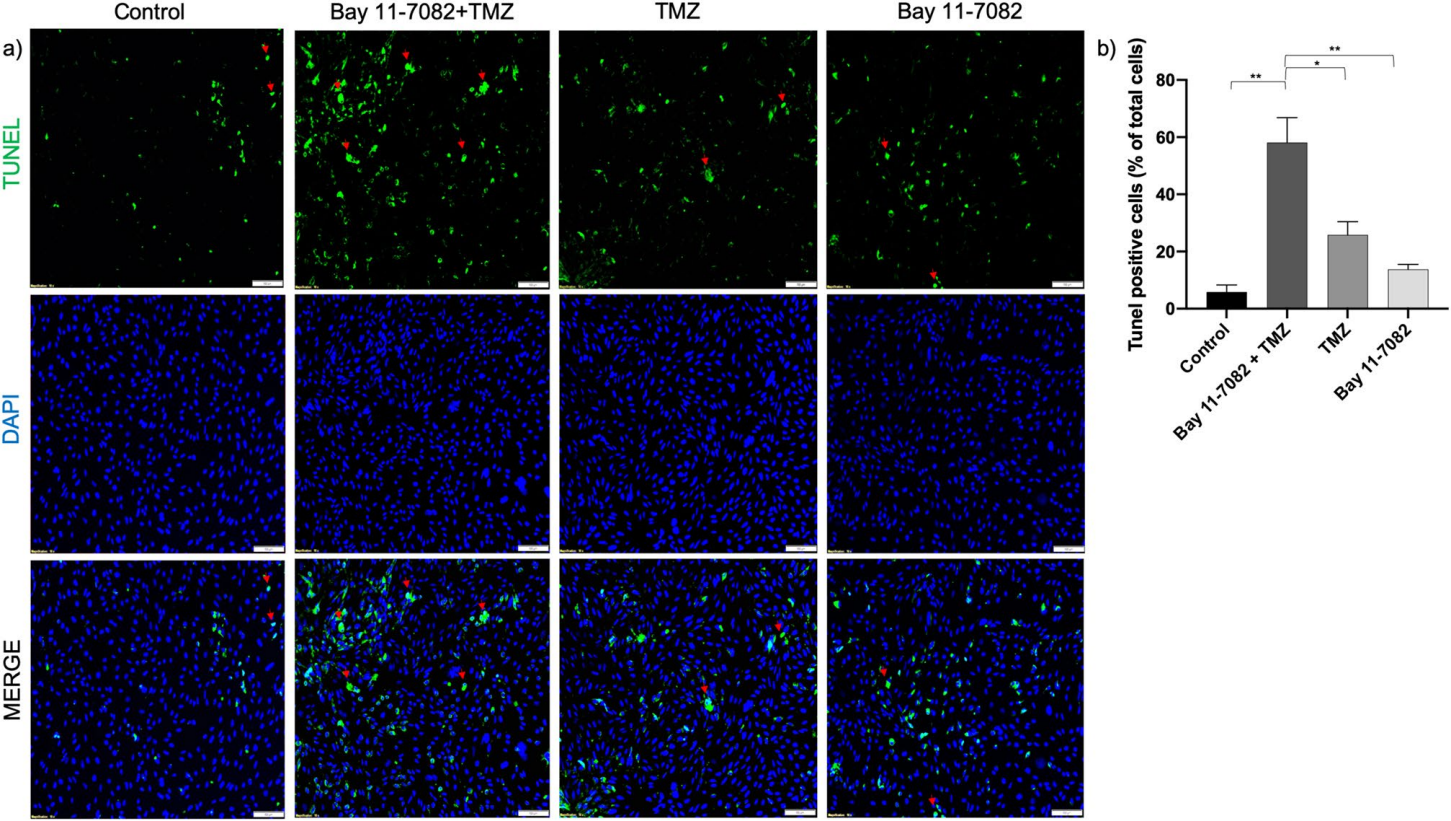
Apoptosis assay (TUNEL). (**a**) Fluorescent images of TUNEL ( +) cells in patient-derived GBM cells. TUNEL assay was performed on cells treated with Bay 11-7082 and TMZ in combination or alone in the microwells. Cells were collected from the microwells, trypsinized and replated into 8-well chamber slides. TUNEL ( +) cells (green) with ring-like nuclear stain are indicated with red arrows. Nuclei were counterstained with DAPI (blue). (**b**) Numbers of TUNEL ( +) cells are presented as mean ± SD of three biological replicates. * *p* < 0.05 and ** *p* < 0.01. X20 objective. Scale bars, 100 µm.

To further investigate whether co-treatment of Bay 11-7082 with TMZ can lead to glioma cell apoptosis and to confirm our RPPA and western blot results, we performed apoptosis assay (TUNEL). The patient-derived GBM cells were co-treated with Bay 11-7082 with TMZ or single drug treated and subjected to TUNEL assay to detect DNA damage (Fig. 4a). The results indicated that TUNEL ( +) cells in the co-treatment were increased tenfold compared with control and 4.4 and 2.4-folds compared with TMZ alone and Bay 11-7082 alone, respectively (*p* < 0.05) (Fig. 4b). Additionally, in some TUNEL ( +) cells, we observed a typical ring type chromatin aggregation underneath the nuclear membrane which suggested an early stage apoptosis^51^ (Fig. 4a, red arrows). There were also a few TUNEL ( +) cells that lacked the typical apoptotic ring-like nuclear structure indicating that they were either at a different stage of apoptosis or alternatively undergoing necrosis^52^ that we have not investigated further.

### Co-treatment of Bay 11-7082 with TMZ changed actin organization by inhibiting FAK phosphorylation and cell migration

**Figure 5:**
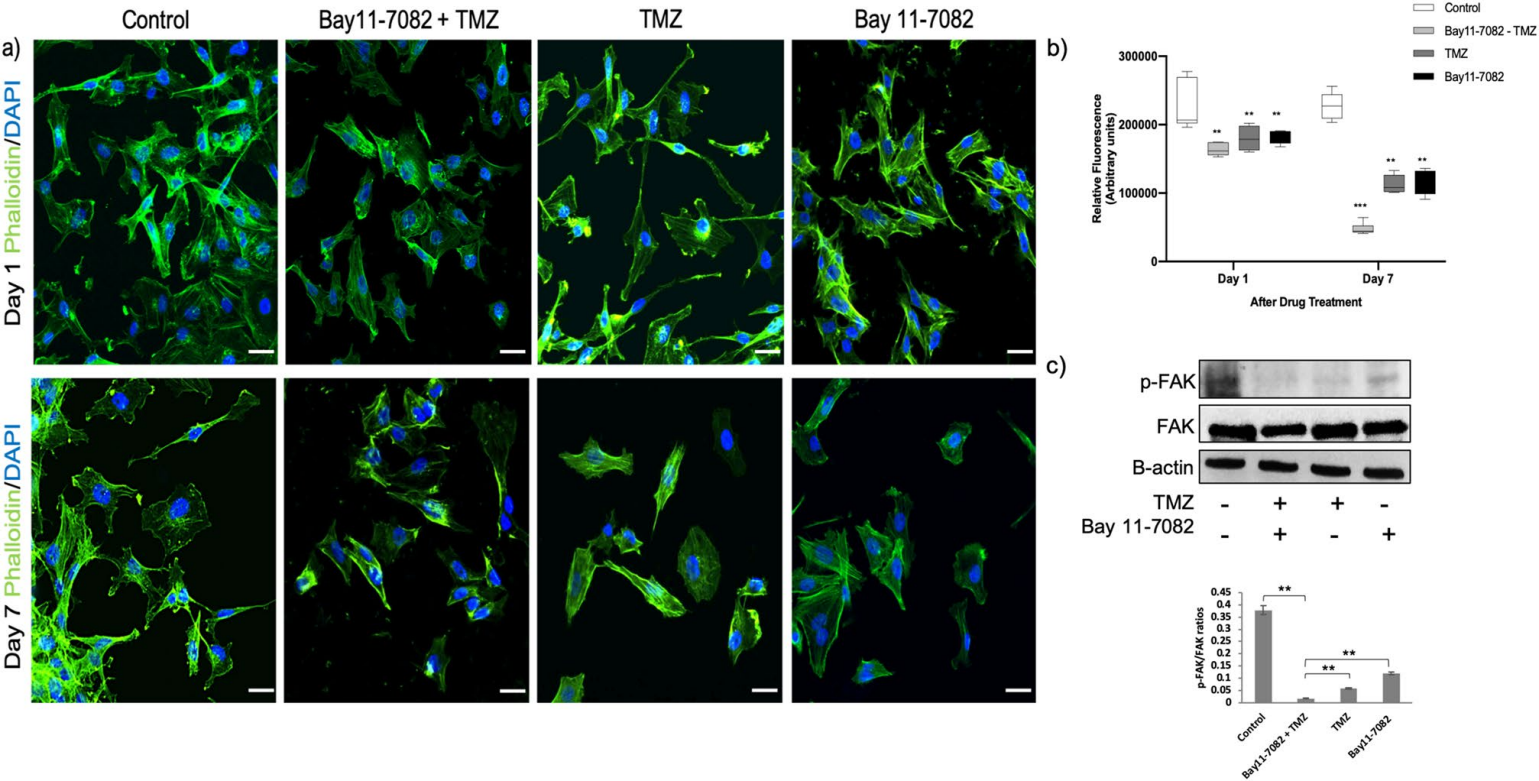
Changes in the actin cytoskeleton and migration pattern in patient-derived GBM cells co-treated with Bay 11-7082 and TMZ or single drug treated in the microwells. (**a**) Upper panel; representative images of the patient-derived GBM cells co-treated with Bay 11-7082 and TMZ or single drug treated at day 1, stained with phalloidin (green) and DAPI (blue). Bottom panel; representative images of the patient-derived GBM cells co-treated with Bay 11-7082 and TMZ or single drug treated at day 7, stained with phalloidin and DAPI. Scale bars, 100 µm. (**b**) Intensity of staining obtained with phalloidin was measured in each cell using ImageJ and displayed as box-plots with 5 to 95% confidence intervals. A two-way ANOVA with Dunnett’s multiple comparisons test was performed to determine statistical relevance. Three biological replicates (n = 30). ** *p* < 0.01, *** *p* < 0.001. (**c**) Representative immunoblots show the levels of FAK pTyr397 and total FAK in patient-derived GBM cell lysates co-treated with Bay 11-7082 and TMZ or single drug treated for 7 days in the microwells. The levels of the proteins were quantified using ImageJ (right panel). Data were normalized to B-actin. Data represent the mean ± SD of three biological replicates. ** *p* < 0.01.

Actin filaments (F-actin) are one of the main components of the cellular cytoskeleton, which regulates actin dynamics and migration process in the cells. The disruption of the actin cytoskeleton inhibits cell migration and adhesion^53^. Depolymerization or cleavage of actin, lamins and other cytoskeletal proteins have been also found to be involved in cell apoptosis^54–56^. To confirm the RPPA results showing changes in the actin modulation pathway and to understand the mechanism that regulates cytoskeletal organization, we treated patient-derived GBM cells co treated with Bay 11-7082 with TMZ or single drug treated. 3D spheroids collected from the microwells were stained with phalloidin (green) and DAPI (blue). Staining cells with fluorescently conjugated phalloidin is considered the most reliable method of accurately labeling F-actin in fixed cells^57^. In the control group, intact cells formed fine-meshed networks with a distinct F-actin organization on both day 1 (Fig. 5a, upper panel) and day 7 (Fig. 5a, bottom panel). In single drug treated cells, actin was still found to be polymerized to filaments as it can be seen by its interaction with phalloidin at both days 1 and 7. However, the cells, which were co-treated with Bay 11-7082 and TMZ lost their F-actin organization and their shape compared with the control and the single drug treated groups at day 7 (Fig. 5a, bottom panel). Changes in the actin distribution within the cells were quantified by measuring the staining intensity using Fiji Macro (ImageJ) as described previously^58,59^. At day 7, we observed a significant decrease in the fluorescence intensity of phalloidin when the cells were co-treated with Bay 11-7082 and TMZ compared with the control and single drug treated groups (*p* < 0.001) (Fig. 5b). To investigate the drug related F-actin mechanism, we examined the levels of FAK protein following co-treatment or single drug treatment. As shown in Fig. 5c, co-treatment significantly decreased the level of phosphorylated FAK compared with both control and single drug applications (*p* < 0.01).

**Figure 6:**
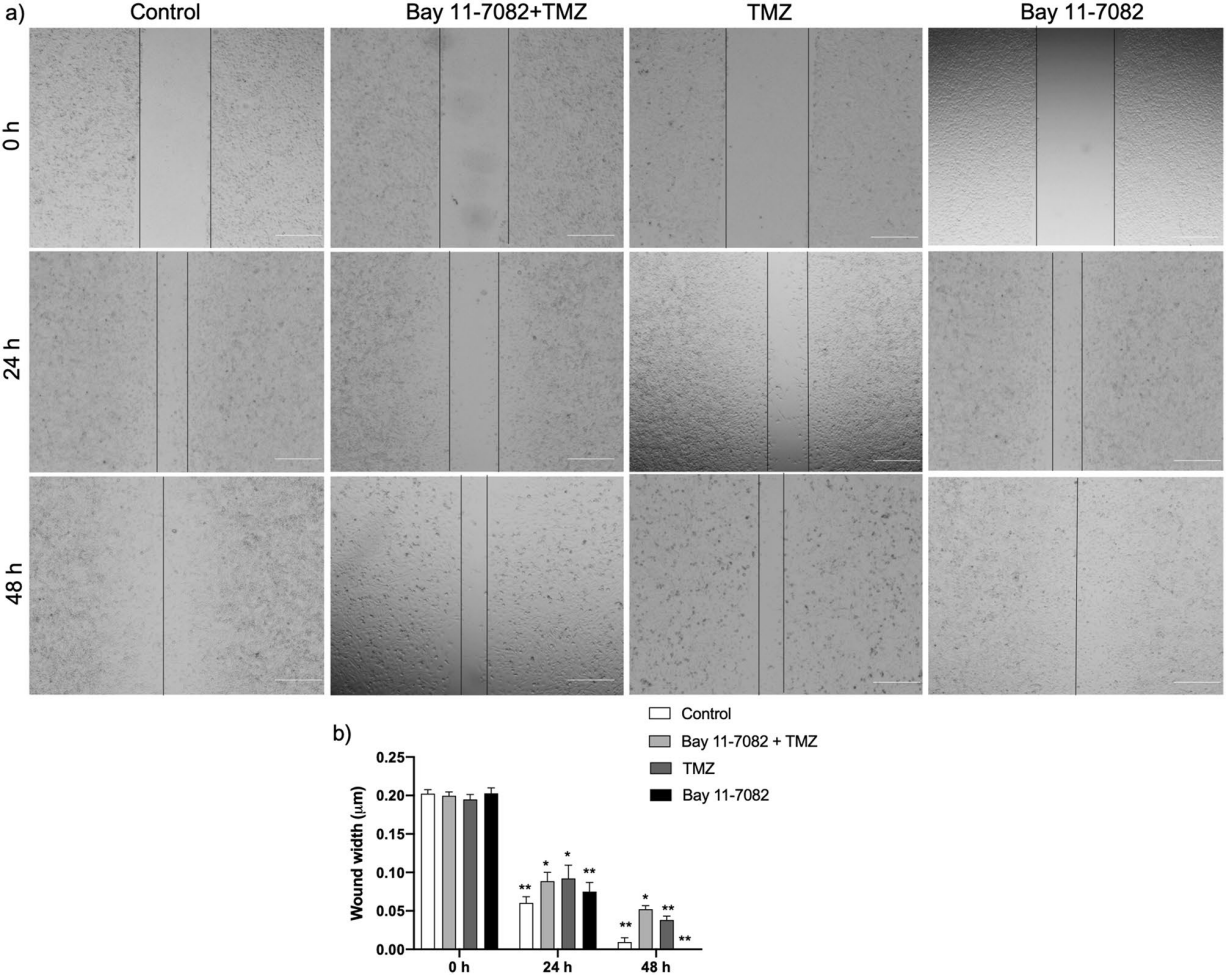
Cell migration of patient-derived GBM cells by wound healing assay. (**a**) Patient-derived cells were co-treated with Bay 11-7082 and TMZ or single drug treated in the microwells, trypsinized and replated in 24-well plates. After they reached to their confluency, a scratch wound was formed with a 200-μl tip and cells were incubated for the next 48 h. Images were taken (4x) at 0 h, 24 hr and 48 hr. Scale bars, 100 µm. (**b**) The wound width was measured with ImageJ and the average wound width was shown. Data represent the mean ± SD of three biological replicates. * *p* < 0.05 and ** *p* < 0.01 (one-way ANOVA with Tukey’s post hoc test).

Furthermore, we investigated cell migration patterns of the patient-derived cells that were co-treated with Bay 11-7082 and TMZ or single drug treated. We collected 3D spheroids from microwells after drug treatment and replated them in 24-well plate to perform scratch wound healing assay. We noted a significant increase in cell density in the scratch area in both control and Bay 11-7082 alone after 24 and 48 h of scratch formation (*p* < 0.01) (Fig. 6a). Although compared with the control cells, both co-treatment and TMZ alone groups showed a decrease in the cell migration into the scratch area after 24 h, we observed that after 48 h, the migration rate of the co-treated cells was significantly slower than the cells that were treated with TMZ alone (*p* < 0.01) (Fig. 6b). These results indicated that the disorganization of actin microfilaments was concomitant with the cell apoptosis after the co-treatment of Bay 11-7082 with TMZ.

## Discussion

Despite the increase in the median survival of GBM patients from 12.1 to 14.6 months^4^, the clinical efficacy of standard of care therapy including TMZ chemotherapy combined with surgery and radiotherapy is still limited. Due to challenges in treating GBM, significant attempts have been made to develop single or combined drug treatments^60–62^. However, given the cost, long time frame, and risks of failure associated with developing a new drug, repurposing available drugs may be the most effective alternative therapeutic strategy. Therefore, it is important to evaluate potential drug combinations for GBM treatment.

Due to the cell repellent property of PEGDA hydrogel, tumor cells can form aggregates at the bottom of the microwells and self-assemble into spheroids in each well within 7 days following cell seeding^31,33,63^. Compared with 2D monolayer cell culture, 3D spheroids have an important advantage: their larger size. Thus, often, drug effects can easily be monitored over time by measuring the size and shape of spheroids^43,44^. Additionally, using 3D in vitro tumor models can better recapitulate in vivo features of the tumors. We used PEGDA hydrogel-based microwell platform^31,33,63^ in order to culture different types of GBM cells, (commercially available GBM cell lines LN229, U87 and patient-derived GBM cells). However, we investigated the effect of the drugs on the patient-derived GBM cells more in detail since growing tumors from tumor biopsy samples could give very detailed information about how tumors respond to drugs^35–37^. Considering the precious nature of the patient samples, this platform, which requires fewer cells compared with 2D monolayer cultures, provides us with a robust tool to recapitulate in *vivo* features of GBM tumors and to test our drug combinations.

NF-κB is one of the major transcription factors associated with GBM and responsible for activating a series of cellular responses, including cell proliferation, survival, invasion and apoptosis^64,65^. Previous studies have shown that NF-κB can activate Akt and promote cell survival and proliferation by down-regulating the expression of phosphatase and tensin homolog deleted on chromosome ten^18,66^. NF-κB pathway can inhibit cell apoptosis by inhibiting a stress-activated protein kinase and a mitogen-activated protein kinase signaling pathway^67^. It can also be activated in response to treatment with cytotoxic drugs, such as vinca alkaloids and topoisomerase inhibitors. Several studies have demonstrated the activation of NF-κB in GBM patient-derived stem-like cells cultures^9,68,69^. Moreover, alkylating agents TMZ can activate NF-κB through DNA damage pathway activation^70,71^. The combination effect of Bay 11-7082 and TMZ have been showed in our previous study where we determined the most effective drug concentrations on GBM cells using our microfluidics platform^42^. Another study that investigated the combined effect of NF-κB inhibitor BAY 11-7082 with TMZ showed that combined drug application induced TMZ resistant in U251 GBM cells^22^. However, the characterization of the precise pattern of NF-κB activation in different GBM cell populations from surgically resected tissues still remains elusive. Therefore, in this study, we investigated the interaction of Bay 11-7082 with TMZ and their effects on the LN299 and U87 GBM cell lines as well as patient-derived GBM cells in order to recapitulate NF-κB activation as in vivo features of the GBM and its signaling pathways. We applied 4.5 µM of Bay 11-7082 and 300 µM of TMZ^34,42^ in combination or alone for all three GBM cell types. First, we observed a significant decrease in both cell viability and size of the spheroids in the co-treatment compared with control and single drug application. Then, we showed quantitatively and qualitatively the expression of NF-κB in all three GBM cell types. We noted a significant decrease in the co-treated group compared with control and single drug application. Our western blot data also confirmed the decrease in the abundance of p-P65, p-P50 and p-IKB-a, that Bay 11-7082 has been shown to inhibit its phosphorylation^46^. However, in the co-treated group, the decrease was significantly higher compared to both control and single drug application. This data showed that co-treatment of Bay 11-7082 and TMZ has more effect on the inhibition of NF-κB pathway than Bay 11-7082 or TMZ alone and suggests a decreased downstream transcription of oncogenic proteins^72^. Although, there were slight differences in the NF-κB expression patterns in three different GBM cell types, we focused on the patient-derived cells in the rest of the study due to their ability to better recapitulate the genomic similarities to primary disease^73,74^.

Proteins that interact with each other activate multiple pathways, which can result in apoptosis according to tissue type and pathological condition. Glioblastoma tumors express high levels of anti-apoptotic BCL2 family proteins such as Bcl-2 and Bcl-xL, which may cause glioblastoma cells to resist apoptosis^75^. The pro-apoptotic members of Bcl2 family, such as Bax and Bak are necessary for their pro-apoptotic effect. Interactions and the ratio between anti-apoptotic Bcl-2 and proapoptotic Bax are decisive factors in the induction of apoptosis^76,77^. Active NF-κB can prevent cells from apoptosis by stimulating the expression of genes and promoting cell proliferation. Although patient-derived GBM samples have been shown to be highly resistant to apoptosis^77^, our data revealed changes in the expression of various members of Bcl2 family and NF-κB signaling pathway after co-treatment of Bay 11-7082 and TMZ. Our RPPA results outlined distinct molecular profiles, in which apoptotic, P53 signaling and NF-κB signaling pathways were significantly affected after the co-treatment. These results supported that the inhibition of NF-κB expression could inhibit the expression of Bcl-2 and promote the expression of Bax thus promote apoptosis. Our data also suggested the possible interaction between Bcl-2 and p53 in regulating cell survival and death^77,78^. The activation of extrinsic and intrinsic molecular pathways can lead to the proteolytic activation caspases. The extrinsic pathway is triggered by proapoptotic ligands that activate cell surface death receptors and procaspase-8, which in turn leads to the cleavage of caspase-3 and apoptosis^79^. Our results determined that the co-treatment significantly inhibited the expression of caspase-3, while the expression of cleaved caspase-3 was increased. Additionally, TUNEL assay, which detects DNA strand breaks which could occur as an event in the apoptosis showed a dramatic increase in the TUNEL ( +) cells after the co-treatment compared with the control and single drug application. Altogether, these results suggested that the inhibition of cell proliferation, Bcl-2 and caspase-3 by the co-treatment of Bay 11-7082 and TMZ may occur through the NF-κB mediated apoptosis and they might be tightly coupled^80,81^.

The literature provides evidence that supports crosstalk between PI3K/Akt/mTOR signaling pathway and NF-κB, which is downstream of Akt. NF-κB activation in GBM regulates through AKT phosphorylation of IκB, resulting in an activated NF-κB that translocates to nucleus^82,83^. Our data showed that when Bay 11-7082 was used with TMZ, there was a decrease in the abundance of PI3K-p110, Akt-pS473, Akt-pT308 and mTOR-pS2448. This preliminary data is important to support the effective use of combined drug treatment to inhibit PI3K/Akt/mTOR/NF-κB signaling pathway.

Finally, alterations in the dynamics of the actin cytoskeleton are critical in determining cell shape and motility, and implicated in cancer cell migration and tumor progression. Many of the cytotoxic agents used to treat cancer patients enhance DNA damage and, to initiate apoptosis in the tumor cells, they promote some morphological changes in the cytoskeleton reorganization. Several reports showed the effect of microtubule-disrupting compounds on NF-κB activation and actin cytoskeleton reorganization^84–86^. Therefore, understanding the regulation of cytoskeleton modulation in GBM is important for developing molecular targets for GBM tumors. Here, we observed a disruption in the actin cytoskeleton modulation associated with the co-treatment of Bay 11-7082 and TMZ. We further confirmed the changes in the abundance of FAK protein and migration pattern of the cells, which may suggest an apoptotic event after a loss of contact to matrix proteins induced by the co-treatment^87^. Thus, these results suggested that actin filament disorganization plays an important role in the Bay 11-7082 and TMZ-induced apoptosis of patient-derived GBM cells.

**Figure 7:**
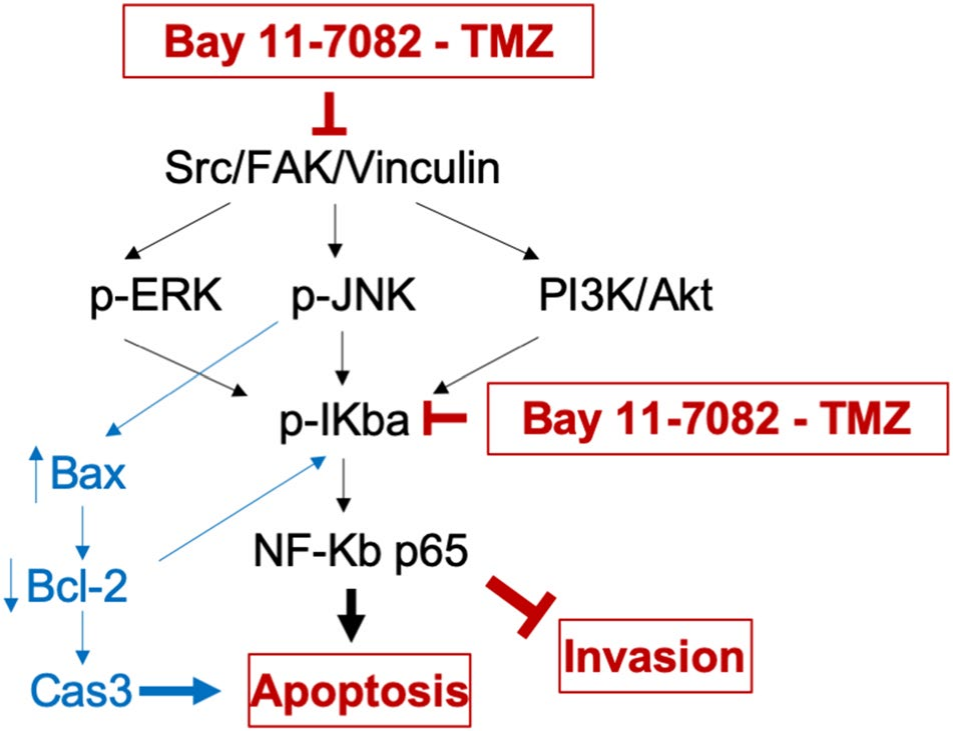
Proposed schematic of the signaling pathways involved in Bay 11-7082 and TMZ-mediated inhibition in GBM patient-derived cells. The effect of combined therapy of Bay 11-7082 and TMZ was achieved through the inhibition of Src/FAK/Vinculin, which regulate the cytoskeleton organization through MAPKs, JNK and PI3K/AKT signaling pathways. Exposure to both Bay 11-7082 and TMZ also leads to receptor-mediated activation of Bax but not Bcl-2 in the subsequent inhibition of the downstream NF-κB transcription factor. Inhibition of NF-κB, in turn, causes cell death.

Taken together, we showed that the co-treatment of Bay 11-7082 and TMZ targeted the function of NF-κB, which potently suppressed GBM proliferation and promoted apoptosis in 3D patient-derived GBM cells. We also showed that the NF-κB signaling pathway was not only related to the cell metabolism, proliferation and apoptotic pathways but also to the modulation of actin cytoskeleton in GBM. Thus, we proposed a potential schematic of signaling pathways involved in the Bay 11-7082 and TMZ-mediated inhibition in GBM patient-derived cells (Fig. 7). Using Bay 11-7082 with TMZ as a therapeutic approach might be an important strategy to achieve the desired effect in GBM therapy.

## Materials and methods

### Drugs and reagents

NF-κB inhibitor Bay 11-7082 and Temozolomide (TMZ) were purchased from Sigma-Aldrich (St. Louis, MO). Antibodies p-p65, p65, p-p50, p50, p-IκB-α, IκB-α, B-actin, Bax, Bcl-2, caspase-3, p-Src, Src, and secondary HRP-conjugated goat anti-mouse antibodies were purchased from Santa Cruz Biotechnology, Inc. (Dallas, TX). Cleaved caspase-3, Bax, p-FAK, FAK and secondary HRP-conjugated goat anti-rabbit antibodies were purchased from Cell Signalling Technology (Danvers, MA). Secondary fluorescence-conjugated goat anti-mouse and goat-rabbit antibodies were purchased from Abcam (Cambridge, MA).

### Cell lines and cell culture

Glioblastoma cell lines LN229 and U87 were purchased from the American Tissue Culture Collection (ATCC) (Manassas, VA). GBM cells were grown in Dulbecco’s modified Eagle’s medium (DMEM) (Gibco, USA) supplemented with 10% FBS (HyClone, USA), 100 U/mL penicillin and 100 g/mL streptomycin (Gibco, USA). We obtained resected glioblastoma tumor biopsies from the UTHealth and Memorial Hermann, Texas Medical Center. The project was approved by both human subject research protection committees at UTHealth and University of Houston, and informed consent for participation in this study was obtained from each subject. All methods were performed in accordance with the relevant guidelines as described previously^34^. The patient-derived GBM tumor cells were cultured in Neurobasal Medium (StemCell Technologies, USA) supplemented with B27, N2, glutamax, heparin sulphate, hFGF and EGF. They were used up to passage 4. All cells were stored in a cell culture incubator at 5% CO_2_, 37 °C.

### 3D spheroid culture in PEGDA microwells

Polyethylene (glycol) Diacrylate (PEGDA) hydrogel-based microwells were prepared using photolithography technique as reported previously^31^. Briefly, to enhance the attachment of PEGDA to the cover glass, glass slides were treated with 3-(Trimethoxysilyl) propyl methacrylate (TMSPMA) and covered with PEGDA solution containing 0.5% (w/w) of the photoinitiator (PI) 2-hydroxy-2-methyl propiophenone for the photopolymerization. PEGDA solution was dropped between a TMSPMA coated cover glass slide and a piece of cover glass with a desired photomask on top for the patterning. To determine the depth of the microwells, cover glasses were used as spacers. The platform was exposed to UV for 30 s at a working distance of 6 inches using the Lumen Dynamics the OmniCure Series 2000 (Lumen Dynamics Group Inc, Canada). Hydrogel droplets were patterned with the 400 µm size photomasks, which were prepared using AutoCAD (Autodesk Inc) and purchased from CADart (Washington, USA).

### Drug administration

After 7 days of spheroid culture in the microwells, co-treatment of NF-κB inhibitor Bay 11-7082 and TMZ or single drug treatment was applied to the cells in the microwells. Bay 11-7082 was dissolved in dimethysulfoxide (DMSO) and diluted to 4.5 µM using appropriate media^42^. TMZ was dissolved in DMSO to prepare a solution of 10 mM TMZ. The solution was then diluted to 300 µM TMZ in appropriate media. Drug administration was done only once, and cells were left in the microwells for 7 days following drug administration. Control (non-drug treated) microwells were maintained under the same conditions.

### Quantification of cell viability

In order to quantify the viability of the spheroids after drug administration, the spheroids treated with drugs and the control group were removed from the microwells, washed with PBS and digested with trypsin. The cells were stained with 0.4% trypan blue solution and counted using a hemocytometer. The viability of the cells in each cell line were normalized to their untreated control group.

### ELISA

To quantitatively detect p-p65 by ELISA, we processed the GBM cells according to manufacturer’s instructions using the Pathscan p-p65(Ser536) ELISA kit (Cell Signaling Technology, Danvers, MA). Briefly, LN229, U87 and patient-derived GBM cells that were cultured in the microwells co-treated with Bay 11-7082 with TMZ or Bay 11-7082 alone, TMZ alone for 7 days. The spheroids were collected from the microwell, washed with PBS and treated with 1X cell lysis buffer. 100 μl of lysate was added per well and the plate was incubate at 37 °C for 2 h. The plate was washed with wash buffer for 4 times. 100 μl of detection antibody was added per well and the plate was incubate at 37 °C for 1 h. The plate was washed with wash buffer for 4 times. 100 μl of secondary antibody was added per well and the plate was incubate at 37 °C for 30 min. The plate was washed with wash buffer for 4 times. 100 μl of TMB Substrate was added per well and the plate was incubate at 37 °C for 10 min. 100 μl of STOP Solution was added per well. The sample concentration was calculated from a standard curve and normalized to total protein content per well evaluated by a spectrophotometer (NanoDrop ND-1000; Thermo Fisher Scientific, Waltham, MA). Control groups without any drug treatment were analyzed simultaneously on each plate for every cell line.

### Reverse-phase protein arrays (RPPA)

We used RPPA to detect activated signaling pathways in patient-derived GBM cells cultured in the microwells and co-treated with Bay 11-7082 with TMZ or Bay 11-7082 alone, TMZ alone for 7 days. Cells were prepared using RPPA lysis buffer to provide the lysates for RPPA analysis by recommended protocols^88^. Briefly, the patient-derived GBM cells were collected from microwells, washed three times in ice-cold phosphate-buffered saline (PBS) and lysed in RPPA lysis buffer (1% Triton X-100, 50 mM HEPES, pH 7.4, 150 mM NaCl, 1.5 mM MgCl2, 1 mM EGTA, 100 mM NaF, 10 mM Na pyrophosphate, 1 mM Na3VO4, 10% glycerol, containing freshly added protease and phosphatase inhibitors (Roche Applied Science Cat. # 05,056,489,001 and 04,906,837,001, respectively)) for 20 min with occasional shaking every 5 min on ice. The resultant solution was centrifuged for 10 min at 14,000 rpm, at 4C. The supernatant was collected, and the protein concentration was determined by BCA protein assay kit (Thermo Scientific) assays and then adjusted to 1–1.5 mg/ml by lysis buffer. Cell lysates were mixed with one-quarter volume of 4 × SDS sample buffer containing 40% glycerol, 8% SDS, 0.25 m Tris–HCl, pH 6.8, and 10% (v/v) 2-mercaptoethanol (freshly added). Samples (n = 6, 3 technical × 2 biological replicates) were sent to the Functional Proteomics Reverse-Phase Protein Array Core (RPPA) facility at the M. D. Anderson Cancer Center for RPPA analysis as described previously^47^. The heat map model was developed by the Department of Bioinformatics and Computational Biology at the M. D. Anderson Cancer Center.

### Immunoblotting

Patient-derived GBM cells were cultured in the microwells, co-treated with Bay 11-7082 with TMZ or Bay 11-7082 alone, TMZ alone for 7 day and collected from the microwell. Then they washed with PBS and lysed using RIPA lysis buffer (20-mM HEPES, pH 7.0, 200-mM NaCl, 1-mM EDTA, 1-mM EGTA, 1% Triton X-100, 5-mM sodium pyrophosphate, 80-mM β-glycerophosphate, 50-mM NaF, 0.1% SDS) including freshly added protease inhibitor cocktail (Sigma-Aldrich), proteasome inhibitor (Calbiochem), and phosphatase inhibitor cocktail 3 (Sigma-Aldrich). Cell lysates were incubated on ice for 30 min and centrifuged at 4 °C, 14 000 rpm for 10 min. Supernatants were collected and the concentration of protein was measured using Bradford Protein reagent (Bio-Rad). Equal amounts of cell lysates were subjected to 4%–20% SDS-PAGE gels, transferred to a PVDF membrane (Thermo Fisher Scientific). Membranes were blocked with 5% milk (in 1 × PBS-Tween20) for 30 min, followed by primary antibody incubation overnight at 4 °C. After three washes with PBS-Tween20 (5 min each), membranes were incubated with secondary antibody (5% milk in PBS-Tween20) for 60 min. Protein bands were visualized using ECL western blot detection system (Amersham Pharmacia Biotech). The data were normalized to B-actin.

### In situ apoptosis assay (TUNEL)

Patient-derived GBM cells cultured in the microwells were co-treated with Bay 11-7082 with TMZ or Bay 11-7082 alone, TMZ alone for 7 day. They were collected from the microwells and trypsinized to assess DNA fragmentation with TACS® 2 TdT-Fluor In Situ Apoptosis Detection Kit (R&D Systems, Inc., Minneapolis, USA) following the manufacture’s instruction. Labeled cells were examined using fluorescence microscopy (Olympus, Tokyo, Japan) using a 405∼488 nm filter.

### Wound healing assay

Patient-derived GBM cells cultured in the microwells were co-treated with Bay 11-7082 with TMZ or Bay 11-7082 alone, TMZ alone for 7 day. The spheroids were collected from the microwells, trypsinized and seeded at a density of 5 × 10^4^ cells per well in 96-well plates in complete cell culture medium. The monolayer of cells was scratched with a 200 μl plastic pipette tip to create a uniform wound. The wound width was then examined after 0, 24 and 48 h of incubation under a phase-contrast microscope at X10 magnification (Olympus, Tokyo, Japan). Images of at least three random fields were taken, and the cell migration ability was expressed by the open wound width.

### Fluorescence staining of F-actin

Patient-derived GBM cells cultured in the microwells were co-treated with Bay 11-7082 with TMZ or Bay 11-7082 alone, TMZ alone for 7 day. The spheroids were collected from the microwell, washed with PBS and cultured on glass slides to sub*-*confluency. Cells were fixed in 4% paraformaldehyde for 10 min, washed 3 times with PBS for 5 min and incubated with phalloidin-conjugate working solution 1 h at room temperature. Cells were rinsed 3 times with PBS for 5 min and mounted with mounting media to preserve fluorescence. Cells were imaged using an Olympus fluorescence microscope (Olympus, Tokyo, Japan). Changes in actin distribution within the cells were quantified by measuring the staining intensity using Fiji Macro (ImageJ) as described previously^58,59^.

### Statistical analysis

Unless otherwise noted, all reported results were from three independent experiments performed in triplicate. Statistical comparisons between groups were performed using the unpaired two-tailed Student’s t-test unless otherwise specified. *p* value < 0.05 was considered to indicate the statistically significant difference between values. The data were presented as the mean ± standard deviation.
